# Evaluation of a Modified Bit Device to Obtain Saliva Samples from Horses

**DOI:** 10.3390/vetsci8100232

**Published:** 2021-10-15

**Authors:** Aviva Vincent, Robin Marie Peth-Pierce, Meghan A. Morrissey, Mary C. Acri, Fei Guo, Lauren Seibel, Kimberly E. Hoagwood

**Affiliations:** 1 Fieldstone Farm Therapeutic Riding Center, 16497 Snyder Rd, Chagrin Falls, OH 44023, USA; 2 Veterinary Social Work Certificate Program, College of Social Work, University of Tennessee (Knoxville), 1618 Cumberland Ave, 401 Henson Hall, Knoxville, TN 37996, USA; 3 Public Health Communications Consulting, LLC., 16678 State Rd., North Royalton, OH 44133, USA; r.pethpierce@vikes.csuohio.edu; 4 Department of Child and Adolescent Psychiatry, NYU Langone Health, New York University (NYU), One Park Avenue, 8th Floor, New York, NY 10016, USA; Meghan.Morrissey@nyulangone.org (M.A.M.); Mary.Acri@nyulangone.org (M.C.A.); Lauren.Seibel@nyulangone.org (L.S.); Kimberly.Hoagwood@nyulangone.org (K.E.H.); 5 Division of Biostatistics, NYU Langone Health, New York University (NYU), One Park Avenue, 8th Floor, New York, NY 10016, USA; Fei.Guo@nyulangone.org

**Keywords:** equine welfare, validation, feasibility, saliva collection, stress response

## Abstract

(1) Background: Accounting for the well-being of equine partners is a responsibility of those engaged in Equine-Assisted Services (EAS). Researchers took heed of this call to action by developing an innovative way to collect data to assess the physiological indicators of stress in equine participants. The collection of saliva is considered to be a minimally invasive method of data collection and is typically performed using a cotton swab; however, in equines, the introduction of a foreign object may induce stress; (2) Methods: Researchers used a modified bit to collect pooled saliva in an effort to further reduce stress during the saliva collection process. Additionally, the collection of pooled saliva, via the bit, increases the opportunity to consider additional analyses, such as oxytocin, which is more reliable in pooled saliva than site-specific saliva captured with a swab; (3) Results: A data analysis demonstrated that ample saliva was captured using the modified bit. Observational data supported that the horses demonstrated fewer physical stress signals to the bit than to the swab. Thus, the modified bit is a feasible and valid method for equine salivary sample collection; (4) Conclusions: The results suggest that the modified bit provides a viable method to collect equine saliva and supports national calls to prioritize animal welfare analysis, specifically for horses used within EAS. Future research should enhance methodological rigor, including in the process and timing, thereby contributing to the bit’s validation.

## 1. Introduction

A growing level of research in equine-assisted services (EAS) demonstrates the impact of horse–human interaction on a range of children in reducing stress and anxiety [1]. As financial investments in animal-assisted intervention and EAS research infrastructure grow [2], so do calls to place an equal effort in assessing the welfare of the *equine* participants in these services [3]. Although EAS studies assess the therapeutic impact of interventions on the participants under study, these studies should also measure the impact on the horse [4]. Welfare can, and often is, measured by observing horse behaviors. Common observable behavioral signs that a horse is stressed include head tossing and shaking, weaving, pinning their ears back, tail swishing, hooves pawing the ground, cribbing, or elimination; less commonly measured signs are the physiological indicators that register stress in the equine’s body [5]. However, there have been no studies conducted to date that assess the accuracy of reports provided by human service professionals on the welfare of a horse, especially in EAS [4].

Human and equine research seeking to understand the internal impact of an intervention tends to rely on measuring indicators of stress, such as cortisol. Cortisol has become a foundational analyte in the process due to its reliability, validity, ease of collection and the cost-effective nature of using Cortisol in testing. Cortisol is a naturally occurring hormone in equines and humans that provides a similar stress response in both. As has been found in emerging data, equines and humans tend to produce cortisol in reaction to stress induced situations in similar timeframes—with a natural diurnal rhythm, and potential peaks around 10 min of an antecedent [6,7,8,9,10]. Other analytes have been included alongside cortisol, such as alpha-amylase, a surrogate indicator of stress release [11]. However, oxytocin—thought to measure relaxation and often referred to as the “happiness hormone”—is the first indicator used to measure positive physiological change; the antithesis of cortisol. With the ease of a single saliva collection—as the analysis requires only 0.5 mL of saliva—the complexity of physiological changes can be measured to understand the stress and relaxation that may result from interactions between humans and horses.

Salivary samples are minimally invasive tools used to assess physiological changes and mechanisms of change between horses and humans. Furthermore, as a quantitative measure, salivary analysis improves scientific rigor and furthers the depth of understanding [7,10]. In behavioral and social sciences, there is an increasing focus on the utilization of saliva data points to supplement other non-physiological (i.e., behavioral health) measures both clinically and diagnostically, as well as in the biomedical research infrastructure [12]. By integrating physiological feedback in addition to self-report, observational and survey data, researchers are able attend to the demand of providing empirical and quantitative data regarding equine welfare and horse–human interaction. Current EAS research that involves collecting equine physiological indicators is conducted via the collection of blood, which is most often used to detect, and best records acute changes in cortisol. However, the process of drawing blood is stressful for horses. Urine is also used to test physiological indicators, but its collection time, ability, and its convenience is inconsistent. Thus, salivary collection and assays are a preferred method for cortisol, and several other physiological, indicators.

Alongside this interest, there has been an increase in scientific knowledge regarding how saliva analytes explain physiological experiences [13]; for example, salivary cortisol can support data about an animal or person’s observed stress behaviors. Assays are an asset in research, especially when combined with significant advances in diagnostic tools, access and availability of supplies, and for quality testing services from the growing list of vendors who support clinical research in this specific international field [14]. Traditionally, saliva samples have been collected utilizing cotton swabs; the swab was inserted into the horse’s mouth along their cheek and held in place for a specified amount of time. Horses often recognize the swab as a foreign object and seek to immediately remove the material from their mouth by raising their head, projecting their tongue, and turning away; all of which are stress responses [5]. Additionally, a swab laid against the side of the cheek collects saliva from specific glands, thereby preventing the collection of a true representation of pooled saliva.

The aim of this study was to assess if this modified bit would reliably collect a sufficient quantity of saliva for analysis of physiological indicators (e.g., oxytocin, cortisol and alpha-amylase). Though three specific analytes were of interest and further assessed in an evaluative study in the subsequent phase of this research, cortisol was included at this time as it is the most prevalent analyte used in current equine-related research.

*Development of an Equine-Friendly Saliva Collection Bit.* A 10-week adaptive horseback riding protocol, called “Reining in Anxiety” (RiA), that combines evidence-based practices for treating childhood anxiety (i.e., cognitive-behavioral elements) with progressive horsemanship skills was created. The intervention posits that the horse is a vital element in delivering the intervention [15]. To explore the horse–human relationship, saliva was collected from equines, riders, and volunteers to assess the trio’s stress response level to the RiA intervention, by analyzing several key physiological indicators, including cortisol, alpha-amylase, and oxytocin. As a precursor to this research, the team recreated a modified bit to evaluate whether it could collect an adequate amount of saliva and thereby act as a reliable method to assess the analytes in question. The first step of evaluation of the modified bit is reported herein; the results of the full RiA intervention in which this bit was used are reported in a separate forthcoming manuscript.

A minimally invasive salivary bit collection method has been developed and piloted to reduce the stress on the study horses by a team of researchers in Murcia, Spain. Contreras-Aguilar and colleagues at the University of Murcia (Spain) developed a novel bit to aid in the collection of saliva [6]. In this initial study, Contreras-Aguilar et al. [6] demonstrated that the modified bit successfully captured ample pooled saliva to reliably test a variety of analytes, including alpha-amylase and cortisol. While testing salivary oxytocin is relatively novel technology, the modified bit collected the sample in the manner required to include oxytocin. As such, if the bit demonstrates reliability in quantity of pooled saliva capture, researchers may consider including oxytocin as an indicator of relaxation in addition to cortisol and alpha-amylase, which are both biomarkers that are indicative of stress.

## 2. Materials and Methods

The proposed study was approved by the Biomedical Research Alliance of New York (BRANY) institutional review board and the New York University (NYU) Langone Health Institutional Review Board.

The research team collaborated with the original creators of the bit [6] to verify detailed aspects of developing the bit, such as length, width, and the placement of the slit through which equine saliva would enter the bit; there were no specified criteria to ensure that the bit was identical. A modified bit based on this already existing internationally-developed design, was created by Fieldstone Farm staff to capture the equine saliva samples for analysis by Salimetrics, Inc. (Carlsbad, CA, USA).

**Figure 1:**
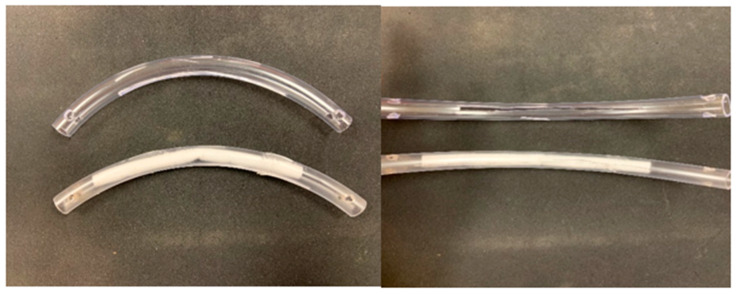
The Modified Bit Developed at Fieldstone Farm (side and front views).

**Figure 2:**
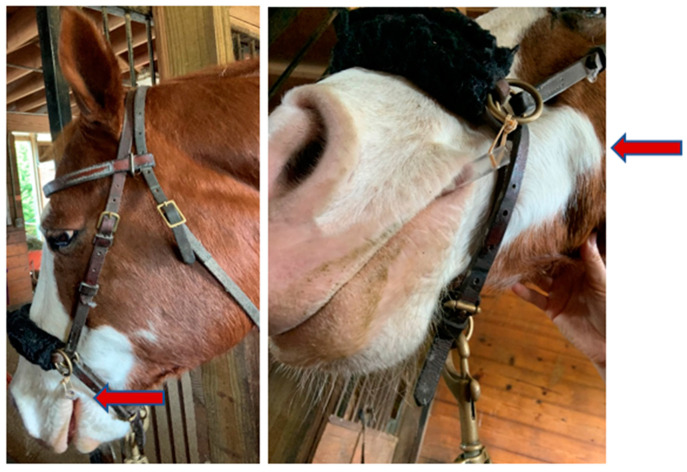
The Modified Bit Attached to The Bitless Bridle for Collection (side and front views).

The bit was created using 10 mm polyvinyl chloride (PVC) tubing, cut to 0.15 m, with a 0.11 m by 0.0025 m wide slit laterally on the rough side (towards the horse’s throat). The bit was secured to the cheek piece of a bitless bridle, to mimic a traditional bit. A 0.05 m by 0.005m gauze was placed in the bit at the time of each collection (see Figure 1 and Figure 2).

Saliva samples were collected using this modified bit prior to the first week of the RiA study to assess their effectiveness in collecting the necessary volume for analysis. Samples were collected for eight horses while they were in their stall. The bit and swab were collected by a researcher on the team (also co-investigator), and supervised by the equine director; where appropriate, the equine director supported the collection, as she is in daily physical contact with each horse. This is in alignment with the study’s aim, which is to collect volume, not to test interaction. The first saliva sample collection used the modified bit; the second set of saliva samples that were collected used the cheek swab. No more than one minute elapsed between the time the bit was removed and the swab was inserted. The order of the bit and swab was chosen by the equine director and researcher through an understanding of the horse’s typical routine. The horses were commonly in lessons during the time of testing. Therefore, being asked to accept a bit would have been in accordance with their routine, whereas if it was an off-hour for medication it would have differed from their routine. While it is possible that the order of bit and swab impacted the horses’ stress level, the aim of this study was to collect sufficient saliva, which was not impacted by the order in which they were administered.

Both of the methods for the bit and the swab, collected saliva for a 90-s interval. After 90 s, the samples were then then placed in appropriate cryovials, and remained frozen (at or below −20 Celsius to minimize degradation and to prevent bacterial growth), per Salimetrics protocol [16]. All samples were shipped overnight, on dry ice, to Salimetrics Inc. (Carlsbad, CA, USA) for assay analysis.

## 3. Results

### Validity of Samples for Analysis

All sixteen cheek swabs (n = 8) and bit (n = 8) samples were valid for analysis, in that they were successfully collected from the horses, with the horses’ willingness to accept the swab and/or bit, keep it in their mouth without demonstrating signs of stress, and to remove it after 90 s. Seven out of eight samples had a sufficient volume of saliva to be valid for analysis; one bit sample collected saliva, but not enough to test in duplicate because while the volume was originally sufficient, after the centrifuge, the remaining substance was not sufficient. The volume may have been composed of water, phlegm, hay material, or other matter.

A paired-samples analysis of these samples showed that the average volume of saliva collected by the cheek swab (1187.50 µL) was higher than the average volume collected by the bit (587.50 µL). While a lower volume was collected by the bit, there was, however, sufficient volume to assess the desired analytes. These results support the feasibility of the modified bit as a viable method to collect equine samples in the larger pilot replication study in which it was used.

The raw data results from the bit (0.287 µg/dL) garnered a lower cortisol reading compared with the swab (1.07 µg/dL) (see Table 1). Observational data supported the data in that horses were required to stand still for the swab collection which often pinning their ears back, whites of eyes, pinched nostrils, and/or tongue thrusting—all of which are signs of physical or environmental stress [7]. With the bit, horses maintained a relaxed body position with neutral ears, soft eyes, and typical nostrils; often, horses ruminated or mouthed the bit as they would do with a typical bit. No discomfort was observed during the use of the bit.

## 4. Discussion

The results suggest that the bit method is a viable way to collect equine saliva. The apparatus should be adjusted to allow for its appropriate fit in the horse’s mouth and an ample flow of saliva. From an equine welfare perspective, the bit may be a more humane alternative; the horses were observationally less stressed when using the bit compared to the swab, which was supported by the raw data. However, it is also possible that this is an artifact of the saliva collection method; perhaps the swab collected more cortisol because of the specific placement along the jaw line, closer to where the cortisol is released. Limitations of the current study include a small sample size, which consistently offered the bit to the horses first, followed by the swab. Additionally, a validated measure could have been used to observe and capture data on the horse’s behavior to the introduction of the bit and the swab to increase the rigor of the findings.

Based on the pre-pilot results, the research team decided to elongate (by 0.025 m) and widen (by 0.003 m) the slit, on the rough side of the re-modified bit, to increase the amount of saliva collected in the next phase of the research, thereby increasing the probability that the required amount (0.5 mL) would be consistently collected. The re-modified bit was used in the 10-week intervention of RiA with preliminary evidence supporting the continued reliability of the bit to capture a sufficient amount of saliva, and to yield findings in analyte analysis (paper forthcoming).

The data supports national calls to action to consider the physiological impact of an intervention through saliva sampling and analysis. This call empowers researchers to prioritize animal welfare analysis, specifically for horses within EAS. In this study, the horses are vital collaborators in understanding how the RiA intervention impacts children’s stress levels. Researchers gain a new depth of understanding about the impact of EAS when exploring the triad impact of horse, rider, and volunteer; especially, when all three are measured using the same physiological indicators. This study represents the first human-equine interaction intervention that recognizes the value of the horse as a collaborator, to the extent that the impact on the horse is measured by the same mechanisms as the people (e.g., pooled saliva, same analytes). Assessing equine-stress levels acknowledges equine welfare in the provision of EAS. “Animal welfare is ultimately human welfare as the welfare of the animal improves, so does the quality of interaction, and thus the benefits for the human participant” [3]. No studies have looked at whether equine professionals can correctly assess their horse’s welfare [4] and as such, the development of this easy-to-use bit contributes to a toolbox that both researchers and practitioners can use in their work when partnering with equines and accounting for their welfare.

## 5. Conclusions

The results suggest that the modified bit provides a viable method to collect equine saliva and supports national calls to prioritize animal welfare analysis, specifically for horses used within EAS. Access to a minimally invasive method to collect equine saliva will allow for researchers across the equine specific, and the equine assisted services industry to account for a horse’s well-being while providing accuracy and intentionality. EAS researchers could continue to use the bit with the horses at rest, or the bit could be used while the horse is being ridden or worked with on the ground. Researchers should consider the analytes they are interested in measuring, the point in time at which the analytes peak, and the time needed for collection. Most collections require no more than 90 s, which is why pre and post intervention proves to be sufficient, however, there is no reason the bit could not be used during an intervention to collect time-sensitive data. Salivary analysis, in addition to observational data, can provide more comprehensive information regarding the impact of intervention delivery. Future research should enhance the methodological rigor by including comparisons for different methods using random assignment. Studies should also attend to differences in process and timing across different types of approaches, with the ultimate objective being the validation of safe, effective, and robust saliva collection methods.

## Figures and Tables

**Table 1:** Initial Pre-Pilot Sampling of Bit vs. Swab Saliva Collection.

Sample ID	Pre Cortisol	Post Cortisol	Mean Cortisol (µg/dL)	Volume (µL)
01-A	1.330	1.317	1.324	* 1000
01-B	0.176	0.161	0.169	* 200–300
02-A	1.070	0.976	1.023	* 1000
02-B	0.201	0.185	0.193	* 2000
03-A	1.026	0.957	0.992	* 2000
03-B	0.637	0.632	0.635	* 150–200
04-A	1.281	1.259	1.270	* 500
04-B	0.238	qns		* 50
05-A	0.843	0.819	0.831	* 1000
05-B	0.230	0.237	0.234	* 1000
06-A	1.066	1.041	1.054	* 1000
06-B	0.344	0.343	0.344	* 200–250
07-A	1.035	1.020	1.028	* 2000
07-B	0.254	0.253	0.254	* 500
08-A	1.066	1.029	1.048	* 1000
08-B	0.184	0.184	0.184	* 500
Mean Cortisol, Swab (A)			1.07 µg/dL	
Mean Cortisol, Bit (B)			0.287 µg/dL	

Key: A = Swab; B = Bit; * values are approximate.

## Data Availability

Data available on request from the corresponding author.
